# Survival after traumatic brain injury improves with deployment of neurosurgeons: a comparison of US and UK military treatment facilities during the Iraq and Afghanistan conflicts

**DOI:** 10.1136/jnnp-2019-321723

**Published:** 2020-02-07

**Authors:** John Breeze, Douglas M Bowley, Stuart E Harrisson, Justin Dye, Christopher Neal, Randy S Bell, Rocco A Armonda, Andrew D Beggs, Jospeh DuBose, Rory F Rickard, David Bryan Powers

**Affiliations:** 1 Academic Department of Military Surgery and Trauma, Royal Centre for Defence Medicine, Birmingham, UK; 2 Department of Surgery, University Hospitals Birmingham NHS Foundation Trust, Birmingham, Birmingham, UK; 3 Department of Neurosurgery, University Hospital of North Staffordshire NHS Trust, Stoke-on-Trent, Staffordshire, UK; 4 Department of Neurosurgery, Loma Linda University, Loma Linda, California, USA; 5 Department of Neurosurgery, Walter Reed National Military Medical Center, Bethesda, Maryland, USA; 6 National Capital Neurosurgery Consortium, Walter Reed National Military Medical Center, Bethesda, Maryland, USA; 7 Department of Neurosurgery, Georgetown University Medical Center, Washington, DC, USA; 8 Surgical Research Laboratory, University of Birmingham, Birmingham, UK; 9 Center for the Sustainment of Trauma and Readiness Skills, R Adams Cowley Shock Trauma Center, Baltimore, Maryland, USA; 10 Division of Plastic, Reconstructive, Maxillofacial and Oral Surgery, Duke University Medical Center, Durham, North Carolina, USA

## Abstract

**Introduction:**

Traumatic brain injury (TBI) is the most common cause of death on the modern battlefield. In recent conflicts in Iraq and Afghanistan, the US typically deployed neurosurgeons to medical treatment facilities (MTFs), while the UK did not. Our aim was to compare the incidence, TBI and treatment in US and UK-led military MTF to ascertain if differences in deployed trauma systems affected outcomes.

**Methods:**

The US and UK Combat Trauma Registries were scrutinised for patients with HI at deployed MTFs between March 2003 and October 2011. Registry datasets were adapted to stratify TBI using the Mayo Classification System for Traumatic Brain Injury Severity. An adjusted multiple logistic regression model was performed using fatality as the binomial dependent variable and treatment in a US-MTF or UK-MTF, surgical decompression, US military casualty and surgery performed by a neurosurgeon as independent variables.

**Results:**

15 031 patients arrived alive at military MTF after TBI. Presence of a neurosurgeon was associated with increased odds of survival in casualties with moderate or severe TBI (p<0.0001, OR 2.71, 95% CI 2.34 to 4.73). High injury severity (Injury Severity Scores 25–75) was significantly associated with a lower survival (OR 4×10^4^, 95% CI 1.61×10^4^ to 110.6×10^4^, p<0.001); however, having a neurosurgeon present still remained significantly positively associated with survival (OR 3.25, 95% CI 2.71 to 3.91, p<0.001).

**Conclusions:**

Presence of neurosurgeons increased the likelihood of survival after TBI. We therefore recommend that the UK should deploy neurosurgeons to forward military MTF whenever possible in line with their US counterparts.

## Introduction

Traumatic brain injury (TBI) is the most common cause of death on the modern battlefield for coalition military forces.^1–4^ Coalition medical treatment facilities (MTF) in Iraq and Afghanistan are classified by the North Atlantic Treaty Organisation (NATO) into Roles (or echelons).^5 6^ Role 1 provides primary healthcare with specialised first aid, triage, resuscitation and stabilisation.^7 8^ Role 2 MTFs provide enhanced resuscitation with the capability for life-saving surgery. Role 2 facilities are divided into ‘Basic (R2B)’, where damage control surgery procedures can be undertaken, and ‘Enhanced’ (R2E) with additional capabilities and greater resources.^7^ Role 3 MTFs provide all the capabilities of the R2E MTF as well as the capability for specialised imaging and surgery, blood banking and laboratory support. The main US-led Role 3 MTFs were located at Balad (Iraq), Baghdad (Iraq) and Bagram (Afghanistan).^9 10^ The main UK-led Role 3 facilities were Basra (Iraq) and Camp Bastion (Afghanistan).^11^ Additionally, until 2011, the Canadian-led multinational Role 3 MTF in Kandahar (Afghanistan) was augmented by neurosurgeons from the US, UK and other nations, including Denmark and Holland.^12 13^

The US and UK adopted different approaches to the specialty mix of surgeons responsible for treating patients with HI.^14–21^ The US deployed neurosurgeons to specific Role 3 MTF.^4 15^ At the other Role 3 MTFs and all Role 2 MTFs casualties, TBIs were either treated by non-neurosurgeons or were stabilised and tactically evacuated (TACEVAC) to a Role 3 MTF where a neurosurgeon was present. Casualties requiring further management for TBI were evacuated to Germany (Role 4) and some back to homeland USA (Role 5). The use of active duty and reserve neurosurgeons at Role 4, in combination with International Red Cross volunteers, and civilians hired under contract, provided flexibility towards care, with numbers at any particular time varying between locations, reflecting the perceived requirements of that moment. The UK did not deploy neurosurgeons to their MTF apart from two exceptions; the first during the first few months of the Iraq conflict in 2003. The second in May 2007 to Camp Bastion Hospital, Afghanistan, in response to concerns raised following review of several neurosurgical cases.^16^ Specialist neurosurgical capability was not maintained as these concerns were felt to be mitigated by policy to retain neurosurgical skill training for non-neurosurgeons and TACEVAC selected patients to the Role 3 MTF at Kandahar.^14 16 18^

Cranial decompression, preferably through craniectomy, is considered to be within the minimal skillset for NATO military surgeons.^14 22^ In 2018, the US military demonstrated that postoperative mortality was significantly lower when craniectomy was initiated within hours of injury.^23^ Two relevant key performance indicators have been identified by UK Defence surgeons: achieving decompressive craniectomy within 4 hours of blunt HI and debridement and closure of penetrating HI within 6 hours of injury.^14 21^ The basic techniques of decompressive craniectomy are taught to UK military non-neurosurgeons during the Military Operational Surgical Training course.^18^ The US Damage Control Neurosurgery course includes tuition in subtemporal decompression.^15^

Multiple papers from the US and UK have described the management and outcomes of recent military patients^3–6 15 16 19 24–27^; however, direct comparison of outcomes is challenging, as differences in methodology and terminology have been used. Our aim was to compare the incidence, injury types and treatment in US and UK-led military MTF to ascertain if differences in surgical care pathways for patients with HI affected outcomes.

## Methods

The US Department of Defence Trauma Registry (DoDTR) and UK Joint Theatre Trauma Registry (JTTR) databases were scrutinised to identify patients with HI admitted to deployed MTF from 2003 to 2011. Initially, the UK JTTR inclusion criteria were patients triggering trauma team activation, but after 2007, included all patients with trauma who required Strategic Evacuation (STRATEVAC).^28^ The US DoDTR inclusion criteria was admission at Role 2 or Role 3 for a traumatic injury <72 hours old or that led to patient’s death.^29^ Coalition troops were engaged in similar warfare and were using comparable collective and personal armour. Injury mechanisms were recorded as blunt, penetrating and ‘other’ (including blast, not coded for in the US DoDTR). Injury distribution was compared using matching Abbreviated Injury Scale (AIS) codes,^30^ and Injury Severity Scores (ISS) were calculated in the standard manner.^31^ Accurate stratification of severity of HI was challenging as the registries did not record standardised data; therefore, we used the Mayo Classification System for Traumatic Brain Injury Severity to adapt registry datasets to define and stratify TBI.^32^

Mortality was defined by final recorded disposition in the registries (ie, up to Role 5). Died of wounds (DOW) was defined as casualties who died after reaching an MTF.^1^ Death prior to arrival at MTF was defined as killed in action (KIA). The US data were provided with KIA already excluded, and it was actively removed from the UK data. Injury distribution was compared using matching AIS codes.^30^ The US DoDTR and UK JTTR code treatment using International Classification of Disease version 9 (ICD-9) and the Office of Population Censuses and Surveys (OPCS) Classification of Interventions and Procedures version 4, respectively.^4^ For HI, direct comparisons of treatment could be made with the exception of scalp repair, which was described in OPCS but not in ICD-9. An adjusted multiple logistic regression model was performed for intracranial bleeding and moderate/severe TBI using fatality as the dependent variable and treatment in US-MTF versus UK-MTF, undergoing surgical decompression, US versus UK military casualty and surgery by neurosurgeon as independent variables. In order to understand the effect of ISS on outcomes, a multivariate logistic regression model was also undertaken with ISS split into terciles (tercile 1=3–15; tercile 2=16–24; tercile 3=25–75) and used as a covariate in the regression model. Reverse stepwise logistic regression was performed with a p value threshold of <0.05 for inclusion. ORs were determined using a χ^2^ test with Yates’ continuity correction and reported with p values and CI. Data analysis was performed using Stata for Mac V.15.1.

## Results

**Figure 1:**
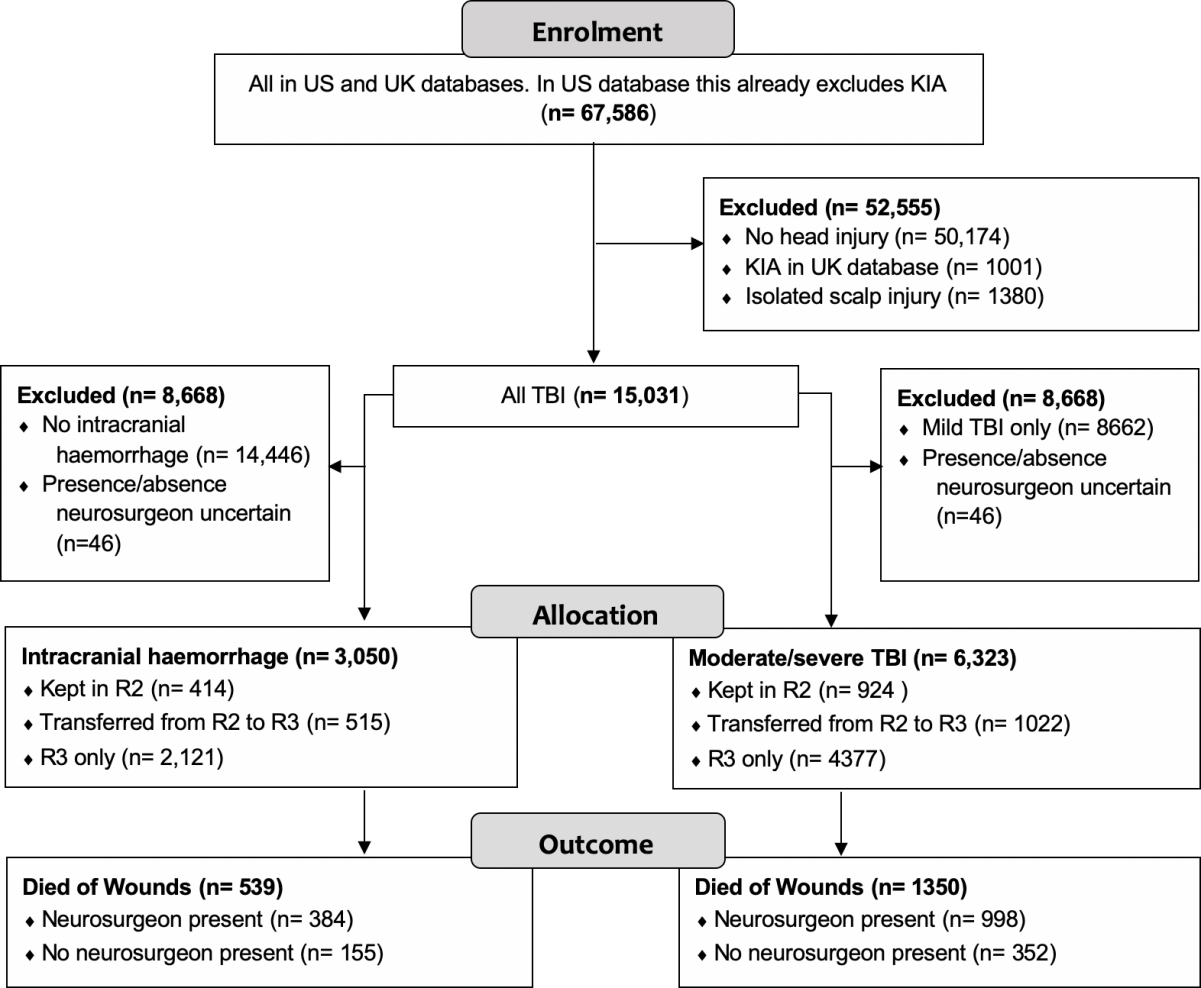
Flow diagram adapted from the CONSORT 2010 reporting template. CONSORT, Consolidated Standards of Reporting Trials; KIA, killed in action.

TBI was present in 15 031/67 586 (22%) of all casualties across both databases, of which 15 737/63 318 (25%) casualties were recorded in the US database (figure 1). Isolated TBI was recorded in 3126 casualties (online supplementary table 1). HI in UK-MTF was typically associated with higher injury severity compared with US-MTF (online supplementary table 1). In univariate analysis, the likelihood of DOW in US-MTF compared with UK-MTF was 1487/14 532 (10%) and 142/499 (28%), respectively (p<0.0001), likely reflecting differences in awareness and recording of mild TBI and the differing criteria for entry into the US and UK combat trauma registries. HI was most commonly from blunt trauma (9289/16 411, 57%) followed by ballistic injury (6740/16 411, 41%, online supplementary table 2). HI most commonly occurred in battle (11 973/16 411, 73%, online supplementary table 3); mechanism of injury included explosion in 57%, motor vehicle incidents (16%) and gunshot wounds (11%). Penetrating TBI was more likely to result in death than blunt (p=<0.0001, OR 3.28, 95% CI 1.47 t0 3.89). TBI due to gunshot was more likely to result in death than from penetrating fragments from an explosion (p=<0.0001, OR 3.98, 95% CI 3.46 to 4.57). Overall mortality for patients with TBI was 1629/15 031 (10.8%). Scalp injuries occurred in 4437/16 411 (27%) of HI (table 1). The most common TBI were skull vault fractures (3379/16 411, 21%) and intracranial haemorrhage (ICH) (3034/16 411, 18%). The most commonly coded treatment for TBI was decompressive craniotomy/craniectomy (1239/1915, 65%) and elevation of skull vault fragments (546/1915, 29%, table 2). Elevation of depressed skull fracture was more likely to be undertaken in MTF where neurosurgeons were present (p=<0.0001, OR 4.38, 95% CI 2.88 to 6.79). In UK-MTF, scalp repair was undertaken on 118/253 (47%) with scalp wounds.

10.1136/jnnp-2019-321723.supp1 Supplementary data

**Table 1:** Anatomical distribution of casualties with head injuries

Group	AIS 2005 diagnosis codes	US military	UK military	Other coalition military	Host nation military	Host nation civilians	All
All head injuries		7349	254	638	2817	5353	16 411
Scalp injury	110099–110808	1606	107	119	930	1695	4437
Intracranial bleed/haematoma	140410–140446, 140629–140656	735	56	86	754	1403	3034
Skull base fracture	150200–150206	632	39	54	381	715	1821
Skull vault fracture	150400–150408	871	68	94	708	1638	3379
Mild concussion (LOC <30 min)	161000–161004	5101	32	331	1335	3527	10 326
Severe concussion (LOC >30 min)	161005–161013	153	6	16	56	107	338
Brainstem injury	140202–140218, 140299	104	51	9	72	140	376
Cerebrum/cerebellum contusion	140402–140626	558	65	58	341	667	1689
Mild TBI		5101	32	331	1335	3526	10 325
Moderate/severe TBI		1558	131	187	1066	2809	5751

Figures include survivors and died of wounds only (killed in action excluded).

AIS, Abbreviated Injury Scale; LOC, loss of consciousness; TBI, traumatic brain injury.

**Table 2:** Treatment performed on patients with head injuries at deployed US-MTF and UK-MTF

Group	ICD-9 procedure codes	OPCS-4 procedure codes	US military	UK military	Other coalition military	Host nation military	Host nation civilians	All
All head (excluding scalp)			517	28	58	429	883	1915
Repair of brain, dura or meninges	02.11–02.13, 02.92, 02.99	A39.2–A39.9	120	6	6	88	221	441
Elevation skull fragment	2.02	V05.3	128	12	12	110	284	546
Craniectomy or craniotomy	01.23–01.25	V03.1, V03.7, V03.8	312	20	35	279	593	1239
ICP monitor placement	01.10, 01.26	A11.3, A20.3	69	3	2	47	71	192

Figures includes survivors and died of wounds only (killed in action excluded).

ICD-9, International Classification of Disease version 9; ICP, Intracranial Pressure; MTF, medical treatment facility; OPCS, Classification of Interventions and Procedures.

**Table 3:** Multiple logistic regression for all casualties with Injury Severity Score as a covariate in the regression model

Survivors	OR	SE	Z	P>z	95% CI
ISS (baseline 0–2)	1	–	–	–	–
ISS on arrival: 3–15	47.23	110.7448	1.64	0.100	0.4768189 to 4678.357
ISS on arrival: 16–24	45.29	106.8278	1.62	0.106	0.4452956 to 4607.922
ISS on arrival: 25–75	0.0196169	0.0449918	−1.71	0.087	0.000219 to 1.757441
Moderate/severe TBI					
Baseline (no/mild TBI)	1	–	–	–	–
Moderate / severe TBI	0.3890276	0.0398801	−9.21	0.000	0.3182159 to 0.4755969
Intracranial haemorrhage (ICH)					
Baseline (no hemmorhage)	1	–	–	–	–
ICH	1.866773	0.1576989	7.39	0.000	1.581921 to 2.202918
Surgical decompression of ICH					
Baseline (no decompression)	1	–	–	–	–
Surgical decompression of ICH	2.356811	0.3035629	6.66	0.000	1.830998 to 3.033622
Neurosurgeon present					
Baseline (not present)	1	–	–	–	–
Neurosurgeon present	3.284088	0.3080936	12.67	0.000	2.732499 to 3.947021

ISS, Injury Severity Scores; TBI, traumatic brain injury.

**Figure 2:**
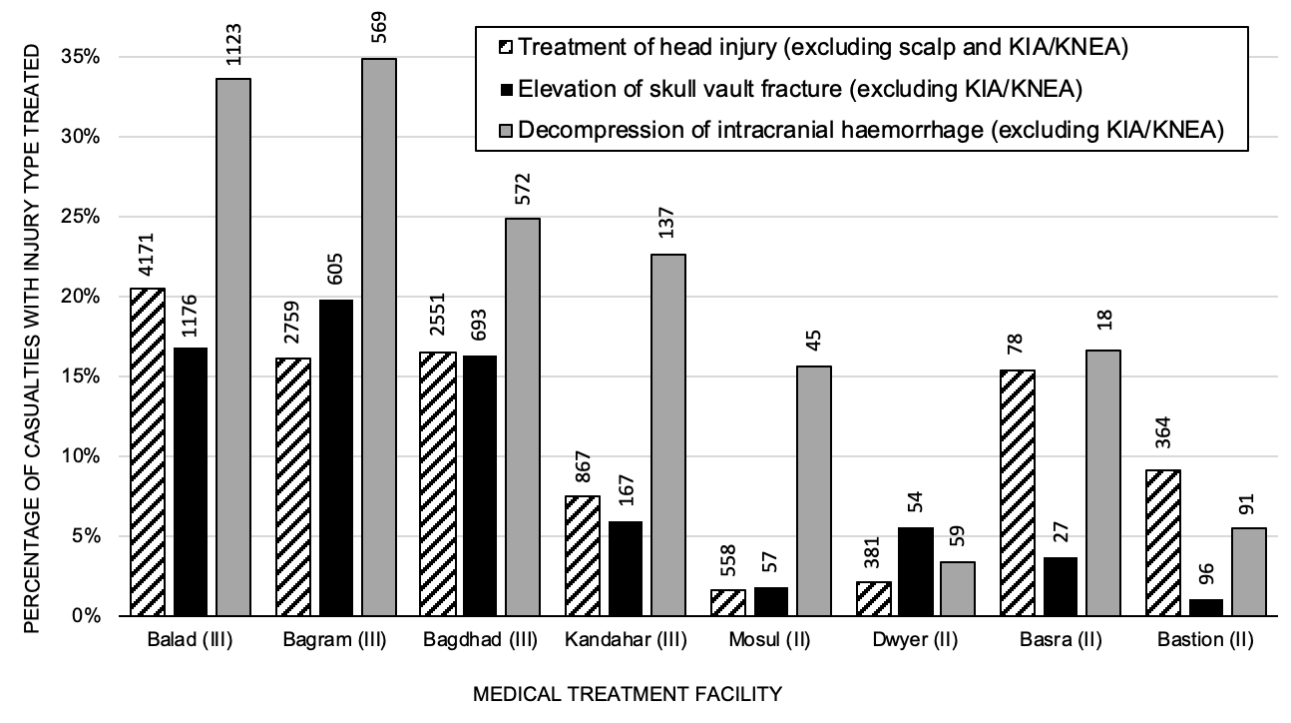
Surgical treatment performed at specific level 2 and 3 medical treatment facilities. All these level 3 facilities had a permanent neurosurgeon, while the level 2 facilities did not. Treatment of head injury excludes scalp repair but includes intracranial pressure monitoring. This graph excludes those killed in action (KIA) and killed non-enemy action (KNEA).

**Figure 3:**
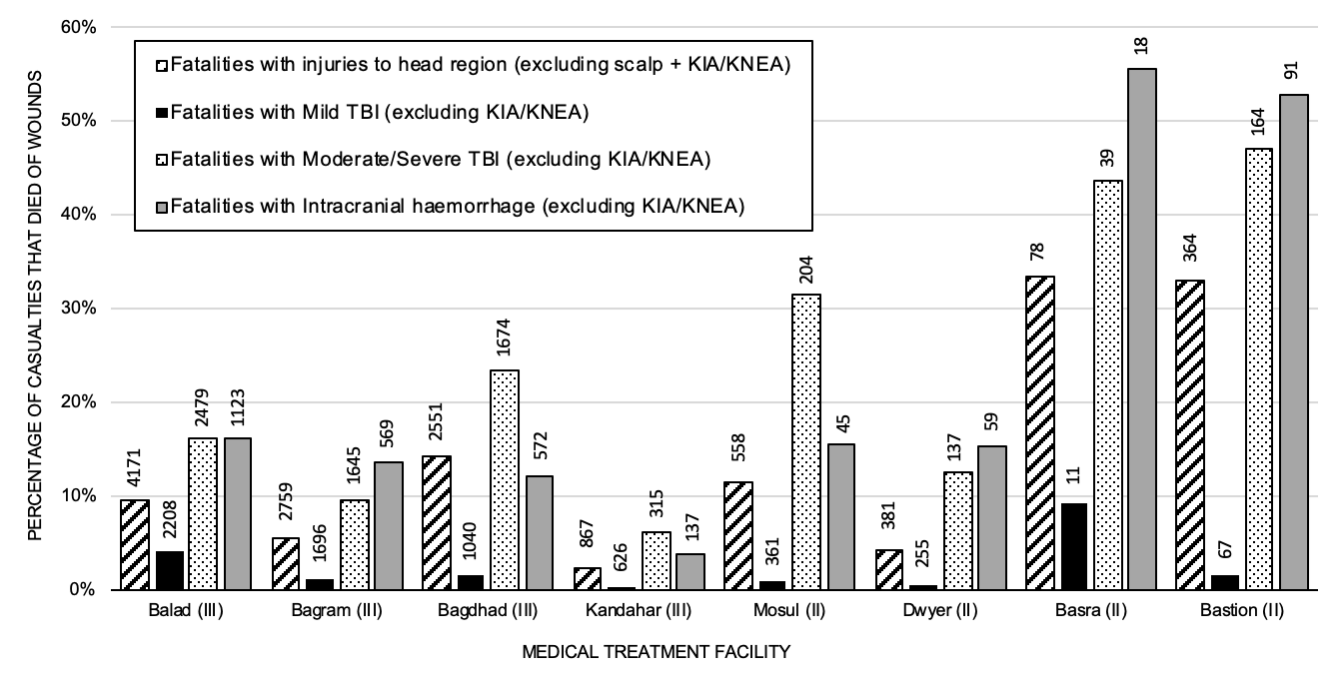
Patients that died of wounds with types of head injury managed at specific level 2 and 3 MTF. All the level 3 facilities had a permanent neurosurgeon, while the level 2 facilities did not. This graph excludes those killed in action (KIA) and killed non-enemy action (KNEA). TBI, traumatic brain injury.

**Figure 4:**
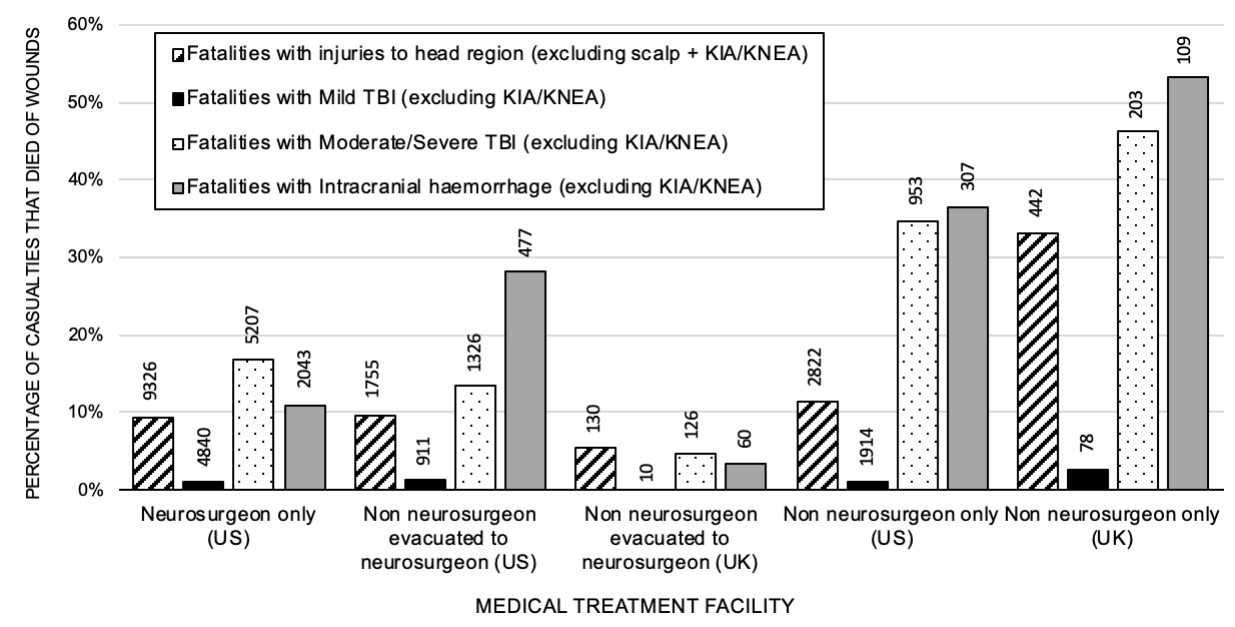
Patients who died of wounds with types of head injury managed at level 2 and 3 MTF demonstrating the effect of aeromedical evacuation to a neurosurgeon on treatment. This graph excludes those killed in action (KIA) and killed non- enemy action (KNEA). TBI, traumatic brain injury.

When analysing casualties with ICH and moderate/severe TBI, multiple logistic regression modelling demonstrated a model with good fit (Area Under the Receiver Operating Characteristic (AUROC) curve=0.70, 95% CI 0.68 to 0.72, model χ^2^=<0.0001). Performing surgical decompression (p=0.013, OR 1.36, 95% CI 1.07 to 1.74) and presence of a neurosurgeon (p<0.001, OR 2.65, 95% CI 2.05 to 3.41) were associated with increased odds of survival in casualties with ICH (online supplementary table 4). Casualties in US-MTF were more likely to have surgical decompression of ICH than in UK-MTF (p=<0.0001, OR 3.44, 95% CI 2.31 to 5.78, figure 2 and online supplementary figure 1). Across both databases, casualties with ICH that underwent surgical decompression were more likely to survive than those that did not (p<0.0001, OR 1.70, 95% CI 1.35 to 2.16, figures 3,4). Surgical decompression for ICH was more likely to be undertaken when a neurosurgeon was present (p<0.0001, OR 7.58, 95% CI 4.64 to 12.70). Casualties with ICH were more likely to survive in MTF where head injuries were managed by a neurosurgeon than a non-neurosurgeon (p<0.0001, OR 3.51, 95% CI 2.80 to 4.39). Analysing the US DoDTR alone, casualties with ICH were more likely to survive in MTF where patients with head injuries were managed by a neurosurgeon than a non-neurosurgeon (p<0.0001, OR 2.90, 95% CI 2.24 to 3.76). Casualties in a UK-MTF with ICH that underwent TACEVAC to the Role 3 MTF at Kandahar (with a neurosurgeon) were significantly more likely to survive than those that were not transferred (p<0.0001, OR 8.34, 95% CI 2.88 to 22.88), reflecting selection of appropriate candidates for specialist intervention.

10.1136/jnnp-2019-321723.supp2 Supplementary data

Across both databases, casualties with moderate/severe TBI were more likely to survive in MTF where head injuries were managed by neurosurgeons (p<0.0001, OR 2.46, 95% CI 2.09 to 2.90), than by non-neurosurgeons (online supplementary table 5). In the US DoDTR, casualties with moderate/severe TBI were more likely to survive in MTF where HI was managed by neurosurgeon than non-neurosurgeon (p<0.0001, OR 2.52, 95% CI 2.14 to 2.97). TACEVAC of casualties with moderate/severe TBI to a neurosurgeon increased survival in both US-MTF (p<0.0001, OR 2.68, 95% CI 1.07 to 3.35) and UK-MTF (p<0.0001, OR 6.27, 95% CI 2.58 to 14.39).

In the simple multivariate model, all three terciles of ISS had no effect on outcome; however, moderate/severe TBI (OR 0.39, 95% CI 0.32 to 0.48, p<0.001) made survival significantly less likely but having a neurosurgeon present made survival significantly more likely (OR 3.28, 95% CI 2.73 to 3.95, p<0.001) (online supplementary table 6). Reverse stepwise logistic regression (AUROC=0.86, 95% CI 0.80 to 0.93, model χ^2^ ^2^ <0.0001) confirmed that the third ISS tercile (ISS 25–75) was significantly associated with a lower survival (OR 4×10^4^, 95% CI 1.61×10^4^–110.6×10^4^, p<0.001); however, having a neurosurgeon present still remained significantly positively associated with survival (OR 3.25, 95% CI 2.71 to 3.91, p<0.001) (table 3). When stratifying head injury severity using the Mayo system and using logistic regression analysis to compare casualties with ICH, US-MTF patients were more likely to survive (p=<0.0001, OR 3.26, 95% CI 2.31 to 5.78) than comparable casualties in UK-MTF. Casualties with moderate/severe TBI treated in US-MTF were more likely to survive (p<0.0001, OR 2.27, 95% CI 1.73 to 2.95) than those in UK-MTF. When patients with isolated HI were analysed, those in US-MTF were also more likely to survive than comparable casualties in UK-MTF (p<0.0001, OR 2.08, 95% CI 1.49 to 2.89). The UK-MTF numbers exclude the 38 casualties with isolated HI that underwent TACEVAC from Camp Bastion to Kandahar for specialist treatment.

## Discussion

This study is the first to directly compare the US and UK combat trauma system databases. Our analysis should be considered in the light of the differing inclusion criteria and data recording within these databases; however, we have found that overall likelihood of survival after combat HI is markedly increased when a neurosurgeon is present in the deployed trauma care system, independent of other risk factors. Combat HI is associated with death and long-term severe disability. In the recent conflicts in Iraq and Afghanistan, the US and UK adopted differing approaches; the US deployed neurosurgeons to many of their Role 3 facilities, whereas the UK did not, relying on forward neurosurgery by non-neurosurgeons or stabilisation by non-neurosurgeons and TACEVAC of selected casualties for neurosurgical care. The use of both active duty and reserve neurosurgeons provided flexibility towards care, with numbers at any particular time varying between locations, reflecting the requirements of that moment. Since the start of the Iraq conflict in 2003, there has been fluctuating numbers of neurosurgeons in both the US DoD and UK MoD that have served on active duty and has varied by service. Future neurosurgery manning for US military medicine in particular is currently under consideration within the broader National Defense Authorisation Act, which is authorised by the US Congress.

According to US registry data, the percentage of casualties with HI doubled in Afghanistan from 30% during 2001–2006 to 59% during 2009–2017^33^ and the 2017 US Joint Trauma System Clinical Practice Guideline for Neurosurgery and Severe Head Injury recommends that: ‘*surgical decompression, or craniectomy, should be strongly considered following penetrating combat brain trauma*’.^34^ This recommendation is generally supported by evidence in this paper; however, the judgement to recognise when to intervene and the technical skills required are complex.^35^ In a report of >100 cranial neurosurgical procedures performed at US Role 2 where neurosurgeons were not deployed, no outcome data were disclosed.^4^ In April 2018, the US DoD published joint trauma clinical practice guidelines for ‘*Emergency Life-Saving Cranial Procedures for non-neurosurgeons*’.^36^ The document includes data from the DoDTR stating that craniectomy procedures were performed by non-neurosurgeons at Role 2 in Iraq and Afghanistan 36 times, with ‘*indeterminate success*’. The discordance in recording of numbers of neurosurgical procedures at Role 2 between the DoD study and other published sources was not examined.

Although cranial decompression is considered to be within the minimum skillset for NATO military surgeons^14 22^ and the basic techniques of craniectomy are taught to UK and US military non-neurosurgeons,^15 18^ neurosurgery is outside the routine daily practice of military non-neurosurgeons.^4^ In 2010, a specialist neurosurgeon recorded his experience in Iraq: he received patients who had been transferred with ‘*malpositioned and inadequate decompressive craniectomies who received no benefits from the operations they had undergone*’; in addition, he noted: ‘*countless medevac missions were created for patients with mild head injuries, frequently placing helicopter personnel at needless risk. At the other end of the triage spectrum, many moribund patients were flown at great risk and cost, only to receive palliative care*’.^15^

Minimising time between critical injury and definitive care has long been a goal of combat trauma systems, and the US and UK used different models. A limitation of this study is that neither combat injury database accurately recorded timelines from injury to care. From 2006 onwards, the UK model comprised provision of advanced resuscitation in the form of the physician-led Medical Emergency Response Team (MERT); US prehospital provision was by paramedic-led Pedro or equivalent. In June 2009, US Secretary of Defense Robert Gates mandated a time standard of 60 min or less from call to arrival at the MTF for transport of US military casualties with critical injuries. A review of >20 000 US military casualties transported by helicopters from 2001 to 2014 demonstrated a decrease in median transport time after the mandate (90 min vs 43 min; p=<0.001) with a reduction in case fatality rate from 3͋7% before to 8% after the mandate (p=0.001).^37^ The UK did not mandate a similar timeline; however, a study involving 975 coalition patients injured in Southern Afghanistan, transported from the point of injury to MTF was published in 2013^38^; the overall mortality for patients transported by MERT and PEDRO was similar (4.2% vs 4.6%, p=0.967). This study has demonstrated low mortality for patients undergoing neurosurgery after TACEVAC from the UK Role 3 hospital in Bastion to Kandahar. However, the conflicts in Iraq and Afghanistan were conducted with coalition air superiority, a circumstance that may not exist in future conflict with peer or near-peer adversaries.

Although most military patients who present with GCS >5 following penetrating intracranial injury survive to discharge,^34^ a study of >1300 injured US military personnel demonstrated, in comparison with veterans with non-head and neck injury, head and neck patients had the highest average disability rating (52%) and the highest proportion of patients rated as 100% disabled.^39^ Mortality is not the only important performance metric in patients with TBI, and scrutiny of longer term functional outcome is essential in assessing results. Accurate prognostication in combat TBI is challenging, particularly early after injury, and high injury severity at presentation is not necessarily associated with long-term poor functional outcome. Of a cohort of UK military patients recorded at presentation to have severe TBI, >70% were in paid employment at 3-year follow-up.^40 41^ To further complicate decision making, with appropriate support, many patients living with severe disablement after TBI express satisfaction with quality of life.^40^

In this study, we present analysis of TBI incidence, patterns of wounding, treatments and short-term outcome for patients arriving alive at deployed US and UK military MTFs from 2003 to 2011. We describe the pattern of injury, treatment and short-term outcome in >15 000 patients with TBI. Coalition databases do not capture standardised data, and longer term outcomes are obscure. The recorded binary outcome of lived or died is insufficient to assess quality of deployed care to brain-injured casualties. A major limitation of this study is that ‘time to event analysis’, such as Cox proportional hazard models or discrete time survival analysis could not be used, as during this time period, time from point of wounding to care data was not routinely recorded in the prospective trauma databases. The implication of this is that immortal time (or survivorship) bias may influence our results; this has been acknowledged, and time is now recorded in the DoDTR and the JTTR. We have identified a significant positive association between survival and the presence of neurosurgeons in deployed military MTFs. This study suggests that overall improvements in military trauma care may have obscured opportunities for improvement in treatment of patients with TBI. We present evidence that can inform future provision of deployed military trauma care and make recommendations about harmonising military trauma registry data capture between coalition partners. In this era of increasing coalition medical interoperability,^7^ standardisation of data capture and recording of longer term functional outcomes would be highly valuable. In this study, presence of specialist neurosurgeons in the deployed trauma care system was associated with increased likelihood of survival after military TBI. We recommend that coalition partners should deploy neurosurgeons to forward military MTF whenever possible.

## References

[1] Eastridge BJ, Hardin M, Cantrell J, et al Died of wounds on the battlefield: causation and implications for improving combat casualty care. J Trauma 2011;71:S4–8. 10.1097/TA.0b013e318221147b 21795876

[2] Keene DD, Penn-Barwell JG, Wood PR, et al Died of wounds: a mortality review. J R Army Med Corps 2016;162:355–60. 10.1136/jramc-2015-000490 26468431

[3] Rosenfeld JV How will we produce the next generation of military surgeons? Re: skill sets and competencies for the modern military surgeon: lessons from UK military operations in southern Afghanistan. Injury 2010;41:435–6. 10.1016/j.injury.2010.01.004 20116057

[4] Turner CA, Stockinger ZT, Bell RS, et al Neurosurgical workload during US combat operations: 2002 to 2016. J Trauma Acute Care Surg 2018;85:140–7. 10.1097/TA.0000000000001915 29965942

[5] Bell RS, Mossop CM, Dirks MS, et al Early decompressive craniectomy for severe penetrating and closed head injury during wartime. Neurosurg Focus 2010;28:E1 10.3171/2010.2.FOCUS1022 20568925

[6] Roberts SAG, Toman E, Belli A, et al Decompressive craniectomy and cranioplasty: experience and outcomes in deployed UK military personnel. Br J Neurosurg 2016;30:529–35. 10.1080/02688697.2016.1208807 27437912

[7] Ministry of Defence Allied Joint Doctrine for Medical Support [Internet]. Allied Joint Publication-4.10(B), 2015 https://assets.publishing.service.gov.uk/government/uploads/system/uploads/attachment_data/file/457142/20150824-AJP_4_10_med_spt_uk.pdf

[8] Bricknell M For debate: the operational patient care pathway. J R Army Med Corps 2014;160:64–9. 10.1136/jramc-2013-000228 24478385

[9] Chan RK, Siller-Jackson A, Verrett AJ, et al Ten years of war: a characterization of craniomaxillofacial injuries incurred during operations enduring freedom and Iraqi freedom. J Trauma Acute Care Surg 2012;73:S453–8. 10.1097/TA.0b013e3182754868 23192069

[10] Powers DB Distribution of civilian and military maxillofacial surgical procedures performed in an air force theatre Hospital: implications for training and readiness. J R Army Med Corps 2010;156:117–21. 10.1136/jramc-156-02-13 20648952

[11] Breeze J, Gibbons AJ, Opie NJ, et al Maxillofacial injuries in military personnel treated at the Royal centre for defence medicine June 2001 to December 2007. Br J Oral Maxillofac Surg 2010;48:613–6. 10.1016/j.bjoms.2009.10.013 19897288

[12] Breeze J, Monaghan AM, Williams MD, et al Five months of surgery in the multinational field hospital in Afghanistan with an emphasis on oral and maxillofacial injuries. J R Army Med Corps 2010;156:125–8. 10.1136/jramc-156-02-15 20648954

[13] Breeze J, Gibbons AJ, Combes JG, et al Oral and maxillofacial surgical contribution to 21 months of operating theatre activity in Kandahar field Hospital: 1 February 2007-31 October 2008. Br J Oral Maxillofac Surg 2011;49:464–8. 10.1016/j.bjoms.2010.08.002 20889245

[14] Breeze J, Blanch R, Baden J, et al Skill sets required for the management of military head, face and neck trauma: a multidisciplinary consensus statement. J R Army Med Corps 2018;164:133–8. 10.1136/jramc-2017-000881 29326127

[15] Teff RJ Use of neurosurgical decision-making and damage-control neurosurgery courses in the Iraq and Afghanistan conflicts: a surgeon's experience. Neurosurg Focus 2010;28:E9 10.3171/2010.2.FOCUS1017 20568949

[16] Eisenburg MF, Christie M, Mathew P Battlefield neurosurgical care in the current conflict in southern Afghanistan. Neurosurg Focus 2010;28:E7 10.3171/2010.2.FOCUS108 20568947

[17] Ramasamy A, Harrisson S, Lasrado I, et al A review of casualties during the Iraqi insurgency 2006--a British field hospital experience. Injury 2009;40:493–7. 10.1016/j.injury.2008.03.028 18656190

[18] Ramasamy A, Hinsley DE, Edwards DS, et al Skill sets and competencies for the modern military surgeon: lessons from UK military operations in southern Afghanistan. Injury 2010;41:453–9. 10.1016/j.injury.2009.11.012 20022003

[19] Rosenfeld JV Damage control neurosurgery. Injury 2004;35:655–60. 10.1016/j.injury.2004.03.006 15203305

[20] Rosenfeld JV A neurosurgeon in Iraq: a personal perspective. J Clin Neurosci 2006;13:986–90. 10.1016/j.jocn.2005.11.013 17056259

[21] Marsden ME, Sharrock AE, Hansen CL, et al British military surgical key performance indicators: time for an update? J R Army Med Corps 2016;162:373–8. 10.1136/jramc-2015-000521 26578479

[22] van Egmond T Report to COMEDS the NATO Military Surgeon ad-hoc Expert Team, MSET, under the auspices of the Emergency Medicine Expert Panel on the development of a minimal skillset for military surgeons deploying to NATO Operations; 2012.

[23] Shackelford SA, Del Junco DJ, Reade MC, et al Association of time to craniectomy with survival in patients with severe combat-related brain injury. Neurosurg Focus 2018;45:E2 10.3171/2018.9.FOCUS18404 30544314

[24] Ragel BT, Klimo P, Martin JE, et al Wartime decompressive craniectomy: technique and lessons learned. Neurosurg Focus 2010;28:E2 10.3171/2010.3.FOCUS1028 20568936

[25] Ragel BT, Klimo P, Kowalski RJ, et al Neurosurgery in Afghanistan during "Operation Enduring Freedom": a 24-month experience. Neurosurg Focus 2010;28:E8 10.3171/2010.3.FOCUS09324 20568948

[26] Breeze J, Gibbons AJ, Shieff C, et al Combat-related craniofacial and cervical injuries: a 5-year review from the British military. J Trauma 2011;71:108–13. 10.1097/TA.0b013e318203304a 21336187

[27] Hawley CA, de Burgh HT, Russell RJ, et al Traumatic brain injury recorded in the UK joint theatre trauma registry among the UK armed forces. J Head Trauma Rehabil 2015;30:E47–56. 10.1097/HTR.0000000000000023 24714212

[28] Vassallo J, Horne S, Smith JE, et al The prospective validation of the modified physiological triage tool (MPTT): an evidence-based approach to major incident triage. J R Army Med Corps 2017;163:383–7. 10.1136/jramc-2017-000771 28739579

[29] Krueger CA, Wenke JC, Ficke JR Ten years at war: comprehensive analysis of amputation trends. J Trauma Acute Care Surg 2012;73:S438–44. 10.1097/TA.0b013e318275469c 23192067

[30] Champion HR, Holcomb JB, Lawnick MM, et al Improved characterization of combat injury. J Trauma 2010;68:1139–50. 10.1097/TA.0b013e3181d86a0d 20453770

[31] Linn S The injury severity score--importance and uses. Ann Epidemiol 1995;5:440–6. 10.1016/1047-2797(95)00059-3 8680606

[32] Malec JF, Brown AW, Leibson CL, et al The Mayo classification system for traumatic brain injury severity. J Neurotrauma 2007;24:1417–24. 10.1089/neu.2006.0245 17892404

[33] Howard JT, Kotwal RS, Stern CA, et al Use of combat casualty care data to assess the US military trauma system during the Afghanistan and Iraq conflicts, 2001-2017. JAMA Surg 2019;154:600 10.1001/jamasurg.2019.0151 30916730 PMC6583837

[34] Smith JE, Kehoe A, Harrisson SE, et al Outcome of penetrating intracranial injuries in a military setting. Injury 2014;45:874–8. 10.1016/j.injury.2013.12.004 24398079

[35] Harvey D, Butler J, Groves J, et al Management of perceived devastating brain injury after hospital admission: a consensus statement from stakeholder professional organizations. Br J Anaesth 2018;120:138–45. 10.1016/j.bja.2017.10.002 29397121

[36] McCafferty RR, Neal CJ, Marshall SA, et al Neurosurgery and medical management of severe head injury. Mil Med 2018;183:67–72. 10.1093/milmed/usy071 30189083

[37] Kotwal RS, Howard JT, Orman JA, et al The effect of a golden hour policy on the morbidity and mortality of combat casualties. JAMA Surg 2016;151:15–24. 10.1001/jamasurg.2015.3104 26422778

[38] Apodaca A, Olson CM, Bailey J, et al Performance improvement evaluation of forward aeromedical evacuation platforms in operation enduring freedom. J Trauma Acute Care Surg 2013;75:S157–63. 10.1097/TA.0b013e318299da3e 23883901

[39] Masini BD, Waterman SM, Wenke JC, et al Resource utilization and disability outcome assessment of combat casualties from operation Iraqi freedom and operation enduring freedom. J Orthop Trauma 2009;23:261–6. 10.1097/BOT.0b013e31819dfa04 19318869

[40] Bahadur S, McGilloway E, Etherington J Injury severity at presentation is not associated with long-term vocational outcome in British military brain injury. J R Army Med Corps 2016;162:120–4. 10.1136/jramc-2014-000393 26385070

[41] Bahadur S, McRann J, McGilloway E Long-term employment outcomes following rehabilitation for significant neurological impairment in UK military personnel: a 3-year study. J R Army Med Corps 2017:jramc-2016-000703 10.1136/jramc-2016-000703 28794008

