# Supplementary material for: Survival after traumatic brain injury improves with deployment of neurosurgeons: a comparison of US and UK military treatment facilities during the Iraq and Afghanistan conflicts
Source: J Neurol Neurosurg Psychiatry. 2020 Feb 7;91(4):359–65. doi: 10.1136/jnnp-2019-321723 (PMC7147183; doi:10.1136/jnnp-2019-321723)
Supplement: Supplementary data [file jnnp-2019-321723supp001.pdf]

	US military treated in US MTF	UK military treated in UK MTF	Other coalition military treated in US MTF	Other coalition military treated in UK MTF	Host nation military treated in US MTF	Host nation military treated in UK MTF	Host nation civilians treated in US MTF	Host nation civilians treated in UK MTF
**Any body area (DOW/DNEA only)**	28935	2013	3965	171	10681	527	19737	1557
**All head (including scalp, DOW/DNEA only**	7349	254	612	26	2709	108	5067	286
**All head (excluding scalp, DOW/DNEA only**	7164	180	556	17	2263	78	4549	224
**Isolated head (DOW/DNEA only)**	1699	101	271	11	918	61	981	120
**Isolated head (excluding scalp, DOW/DNEA only)**	1670	53	118	6	348	31	811	89
**Iraq**	4112 (57%)	49 (27%)	74 (13%)	3 (18%)	1142 (49%)	1 (1%)	2244 (51%)	20
**Afghanistan**	3102 (43%)	133 (73%)	493 (87%)	14 (82%)	1169 (51%)	77 (99%)	2133 (49%)	199
**Mean age (range)**	25·7 (18-60)	25·4 (18-53)	26·9 (18-53)	26·1 (20-43)	26·1 (14-61)	23·8 (12-48)	24·9 (1-85)	19·8 (1-69)
**Male (%)**	7068 (98%)	175 (96%)	561 (99%)	14 (100%)	2311 (100%)	77 (99%)	3852 (88%)	189
**Mean ISS (all head, excluding scalp, DOW/DNEA only)**	10·8	41·6	10·6	39·2	16·1	36·2	15·5	36·2
**Median ISS (all head, excluding scalp, DOW/DNEA only)**	6	34	9	33	14	29	14	29
**Mean ISS (isolated head, excluding scalp,**	4·8	44·1	5·9	40·2	14·1	38·2	14·2	40·4

**DOW/DNEA only)**								
**Median ISS (isolated head, excluding scalp, DOW/DNEA only)**	4	54	4	26	9	26	9	26
**GCS scores 3-8**	937 (13%)	70 (38%)	95 (17%)	5 (29%)	918 (41%)	35 (45%)	1629 (36%)	102 (46%)
**GCS scores 9-12**	141 (2%)	13 (7%)	20 (4%)	2 (12%)	158 (7%)	6 (8%)	363 (8%)	16 (7%)
**GCS scores 13-15**	6086 (85%)	97 (54%)	441 (79%)	10 (59%)	1187 (52%)	37 (47%)	2557 (56%)	106 (47%)
**Fatalities (all head excluding scalp)- DOW/DNEA only**	370/7164 (5%)	46/180 (26%)	25/556 (4%)	5/17 (29%)	319/2263 (14%)	23/78 (29%)	773/4549 (17%)	68/224 (30%)
**Fatalities (isolated head excluding scalp)- DOW/DNEA only**	117/1670 (7%)	14/53 (26%)	15/118 (13%)	1/6 (17%)	106/348 (30%)	11/31 (35%)	252/811 (31%)	29/89 (33%)

Online Table 1 : Demographics of all patients with head injuries. Survivors and Died of Wounds only (Killed In Action excluded).

Mechanism	US military	UK military	Other coalition military	Host nation military	Host nation civilians	All
**Incidence**						
**Blunt (all)**	5268 (72%)	69 (27%)	372 (58%)	1095 (39%)	2485 (46%)	9289 (57%)
**Blunt (explosive)**	3562 (48%)	47 (19%)	226 (35%)	389 (14%)	511 (10%)	4735 (29%)
**Penetrating (all)**	1996 (27%)	181 (71%)	254 (40%)	1633 (58%)	2676 (50%)	6740 (41%)
**Penetrating (GSW)**	393 (5%)	39 (15%)	52 (8%)	458 (16%)	856 (16%)	1798 (11%)
**Penetrating (explosive)**	1564 (21%)	132 (52%)	195 (31%)	1063 (38%)	1531 (29%)	4485 (27%)
**All**	**7349 (100%)**	**254 (100%)**	**638 (100%)**	**2817 (100%)**	**5353 (100%)**	**16411 (100%)**
**Fatalities**						
**Blunt (all)**	157 (42%)	4 (9%)	5 (17%)	87 (24%)	261(33%)	514 (32%)
**Blunt (explosive)**	127 (34%)	2 (4%)	2 (7%)	23 (6%)	61 (8%)	215 (13%)
**Penetrating (all)**	204 (55%)	40 (85%)	24 (80%)	269 (73%)	548 (69%)	1085 (67%)
**Penetrating (GSW)**	109 (29%)	22 (47%)	17 (57%)	140 (38%)	277 (35%)	565 (35%)
**Penetrating (explosive)**	89 (24%)	18 (38%)	6 (20%)	116 (31%)	234 (29%)	463 (29%)
**All**	**370 (100%)**	**47 (100%)**	**30 (100%)**	**369 (100%)**	**795 (100%)**	**1611 (100%)**

Online Table 2: Recorded mechanisms of injury for all patients with head injuries (HI) by mechanism. Includes scalp, survivors and Died of Wounds (excludes Killed in Action).

Mechanism	US military	UK military	Other coalition military	Host nation military	Host nation civilians	All
**All battle**	**5823 (79%)**	**207 (81%)**	**500 (78%)**	**2239 (79%)**	**3204 (60%)**	**11973 (73%)**
**Explosive Device**	5174 (70%)	167 (66%)	426 (67%)	1488 (53%)	2129 (40%)	9384 (57%)
**Gunshot Wound**	393 (5%)	39 (15%)	52 (8%)	458 (16%)	856 (16%)	1798 (11%)
**Motor Vehicle Collision**	133 (2%)	0 (<1%)	4 (<1%)	91 (3%)	133 (2%)	361 (2%)
**Fall**	38 (<1%)	0 (<1%)	3 (<1%)	15 (<1%)	20 (<1%)	76 (<1%)
**Other/not specified/unknown**	41 (<1%)	1 (<1%)	15 (2%)	187 (7%)	66 (1%)	310 (2%)
**All non-battle**	**1526 (21%)**	**47 (19%)**	**138 (22%)**	**578 (21%)**	**2149 (40%)**	**4438 (27%)**
**Motor Vehicle Collision**	761 (10%)	29 (11%)	68 (11%)	126 (4%)	1207 (23%)	2191 (13%)
**Fall**	441 (6%)	7 (3%)	23 (4%)	15 (<1%)	367 (7%)	853 (5%)
**Machinery/ equipment**	101 (1%)	0 (<1%)	10 (2%)	1 (<1%)	44 (<1%)	156 (<1%)
**Other/not specified/unknown**	64 (<1%)	11 (4%)	37 (6%)	436 (15%)	531 (<1%)	1079 (7%)
**All**	**7349 (100%)**	**254 (100%)**	**638 (100%)**	**2817 (100%)**	**5353 (100%)**	**16411 (100%)**

Online Table 3 : Mechanism of injury for all patients with all head injuries. Includes scalp injuries, survivors, Killed in Action and Died of Wounds.

Survival with intracranial haemorrhage	Odds Ratio	Standard Error	z	P>|z|	Significance	95% Confidence Interval	
**Treatment in US MTF**	1·4467	0·347	1·54	0·124	No	0·9040408	2·315131
**US military casualty**	1·0727	0·418	0·18	0·857	No	0·4995449	2·303653
**UK military casualty**	0·4229	0·210	-1·73	0·083	No	0·1597895	1·119711
**Host nation military or civilian casualty**	0·3927	0·144	-2·55	0·011	Yes	0·1914791	0·8054183
**Decompression performed**	1·3628	0·169	2·49	0·013	Yes	1·067778	1·739352
**Neurosurgeon present**	2·6456	0·345	7·47	<0·001	Yes	2·049563	3·414868

Online Table 4: Multiple logistic regression for all casualties with intracranial hemorrhage with the dependent variable being survival.

Survivors with moderate/severe TBI	Odds Ratio	Standard Error	z	P>|z|	Significance	95% Confidence Interval	
**Treatment in US MTF**	1·1194	0·2183359	0·58	0·563	No	0·7637665	1·640627
**US military casualty**	0·7855185	0·1875173	-1·01	0·312	No	0·4919936	1·254161
**UK military casualty**	0·4493001	0·1567288	-2·29	0·022	Yes	0·2267842	0·8901438
**Host nation military or civilian casualty**	0·3973886	0·091345	-4·01	<0·001	Yes	0·2532535	0·6235558
**Neurosurgeon present**	2·461416	0·2042812	10·85	<0·001	Yes	2·0919	2·896204

Online Table 5: Multiple logistic regression for all casualties with moderate or severe TBI with the dependent variable being survival.

Survivors	Odds Ratio	Std. Err.	z	P>z	[95% Conf.	Interval]
Surgical decompression of ICH	2.358124	.3036665	6.66	0.000	1.832118	3.035147
Neurosurgeon present	3.257674	.305056	12.61	0.000	2.711434	3.913957
ISS on arrival: 25-75	.0004324	.0002176	-15.39	0.000	.0001612	.0011596
Moderate / severe TBI	.3886486	.0397897	-9.23	0.000	.3179885	.4750101
Intracranial haemorrhage	1.862212	.1572465	7.36	0.000	1.578167	2.19738

Online Table 6: Reverse stepwise logistic regression of high Injury Severity Score.
