# Supplementary figures and images for: Survival after traumatic brain injury improves with deployment of neurosurgeons: a comparison of US and UK military treatment facilities during the Iraq and Afghanistan conflicts
Source: J Neurol Neurosurg Psychiatry. 2020 Feb 7;91(4):359–65. doi: 10.1136/jnnp-2019-321723 (PMC7147183; doi:10.1136/jnnp-2019-321723)

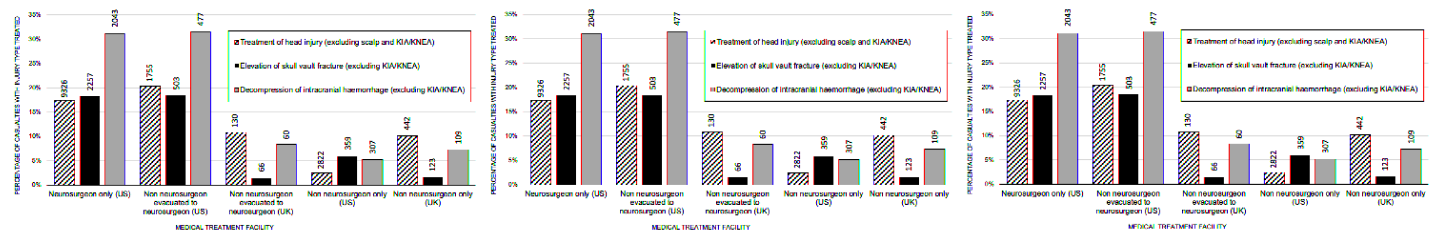

Supplement: Supplementary data [file jnnp-2019-321723supp002.pdf]
